# Economic situation, the key to understanding the links between CEOs’ personal traits and the financial structure of large private companies

**DOI:** 10.1371/journal.pone.0218853

**Published:** 2019-07-18

**Authors:** Jorge Hernández-Pérez, Salvador Cruz Rambaud, Tomás Lorenzana de la Varga

**Affiliations:** 1 Department of Quantitative Methods, University of Loyola Andalucía, Sevilla, Spain; 2 Department of Economics and Business, University of Almería, Almería, Spain; Universidad Loyola Andalucia Cordoba, SPAIN

## Abstract

Executives act based on their experiences, values, personality and personal interpretation of the situations which they face in their companies. Investigations in the field of Behavioral Corporate Finance have determined that there are some relations between CEOs’ personal traits and financial decisions of large companies, but these results are based on indirect personal trait measurements and on public companies. To see whether such relations also exist between CEOs’ personal traits and the financial structure of large private companies, we used psychometric tests to measure their level of optimism, risk attitude and affect heuristic, collected financial data for a period of fourteen years, and considered the economic situation of the country as a key factor in these relations. This paper reports the relationship of executives’ personal traits with the financial structure of large Spanish companies for the period 2001–2014. We observed that executives’ high optimism (and risk aversion attitude) is positively (negatively) related to long-term debt, whilst positive affect is directly associated to the financial leverage and short-term debt. This paper also reports a change of relations when taking into account the country’s economic situation. In effect, by considering this new variable, executives’ risk aversion is seen to be associated to financial leverage and short-term debt, whilst CEOs’ positive affect is linked to long-term debt. These relations are strongly moderated and they become statistically significant in a contracting economic period. In conclusion, the links between CEOs’ personal traits and financial structure of large private Spanish companies make sense when the effect of the economic situation is taken into account. Furthermore, the awareness of these links helps to understand the financial decisions taken within large Spanish companies.

## Introduction

In order to gain a better understanding of corporate decision-making by Chief Executive Officers (hereinafter, CEOs), it had been hypothesized that executives act on the basis of their personal interpretation of the situations which they face, which in turn is based on their experiences, values, and personality, as described by the Upper Echelon Theory [1]. Recently, a set of studies has revealed certain relations between CEOs’ personal traits and company decisions. It has been shown that an executive’s overconfidence is associated with the use of lower discount rates to evaluate cash flows, greater investment and consequently a higher level of borrowing [2]. Moreover, those CEOs who underwent military service during early adulthood tend to take greater risks, resulting in the adoption of a more aggressive capital structure [3]. In addition, narcissistic executives -those with an inflated opinion of themselves and their abilities- are associated with more acquisitive behavior, greater strategic dynamism and more volatile performance [4]. Finally, highly optimistic CEOs are associated with high levels of short-term debts [5].

The investigations in this field usually focus on large public companies and show shortcomings such as indirect CEOs’ personal traits measurement [2] and subjective financial data [5]. Moreover, they disregard other factors which may influence the association of variables such as the economic situation of the country.

To investigate the associations between CEOs’ personal traits and the financial structure of large private companies, we have used a psychometric test to measure executives’ personal traits, and we have collected financial data for a period of fourteen years to explore the links between both types of variables in the long term. In addition, we have taken into account the economic situation of the country (economic expansion and contraction) during the time horizon of the analysis as a key factor for the relationships between variables.

This paper reports the relationship between executives’ personal traits and the financial structure of large Spanish companies for the period 2001–2014. However, it is necessary first of all to define these two parameters before this relationship can be analyzed.

On the one hand, CEOs are characterized by their positive expectations of the future by showing a high level of dispositional optimism. Thus, when dealing with risky financial tasks, they show an attitude of aversion. In contrast, they have a more positive attitude towards these tasks since they feel that the expected benefits are higher than the risks which they perceive as implicit in them.

On the other hand, the financial structure of large Spanish companies usually involves bank debts. In particular, large Spanish companies preferred funding their operations with short-term rather than long-term debt. In the subperiod 2008–2014, the Spanish economy fell into recession and the financial structure of companies showed a slight change in their composition characterized by an increment of long-term debt and a slight diminishing of short-term debt.

The results from the random-effects regression confirm the existence of a relationship between CEOs’ personal traits and the financial structure of large Spanish companies. Moreover, CEOs’ positive affect is directly related to the financial leverage and to the short-term debt of companies. This is because, when they handle short-term debt affairs, they probably expect higher profits and perceive a lower risk of funding. This trait has not been studied in previous investigations. Our results show that executives’ high optimism is positively related to long-term debt, although a direct association with short-term debt is rather to be expected [5] [6]. Although CEOs’ risk aversion attitude is not usually related to company debts [5], our results suggest that it is inversely linked with long-term debt. Due to the fact that these two traits are contradictory with respect to long-term debt, it is likely that CEOs expect a positive future for the company when it is financed with the perceived safety margin offered by long-term debt.

This paper also reports the importance of taking into account the country’s financial position in those periods during which the relationship between CEOs’ personal traits and financial leverage of large Spanish companies is studied. From the regression analysis, several interactions can be observed between the personal traits and the economic situation. From a simple analysis of results, CEOs’ risk aversion is negatively related to the financial leverage and the short-term debt of companies. Furthermore, executives’ positive affect is inversely related to long-term debt. The aforementioned relationships are strongly moderated in both economic periods, but these relationships are only statistically significant during a period of economic recession, as indicated by the results derived from the Johnson-Neyman test.

Despite CEOs’ positive expectations of funding with long-term debt, the level of debt which executives perceive as acceptable to avoid high risks is lower in a contracting rather than an expanding economic period. Furthermore, during the contraction period, they may perceive short-term debt as a more risky option than in a period of expansion, and consequently prefer to reduce it considerably.

The rest of this paper is organized as follows: Section 2 describes the methodology employed to obtain our results, to define the sample, to acquire qualitative (CEO traits) and quantitative (financial structure and company characteristics) data and, finally, to describe the statistical methods used to test our hypothesis. Section 3 presents the results derived from the qualitative and quantitative data and the statistical method applied to them, analyzes the results and puts them in perspective within the related literature. Finally, Section 4 summarizes and concludes.

## Methodology

In this paper, we have constructed a new dataset by merging the balance sheet information for a sample of large Spanish companies with the data obtained from a survey of their CEOs’ personality traits. Firstly, we describe the way of obtaining the list of companies to be studied. Then, we define the qualitative and quantitative variables to be considered in this study. Finally, we describe the tools which have been used to obtain the results.

### Sample

This study examined the data referring to the financial situation of certain companies and their CEOs’ personal traits. First, we determined a set of conditions which they had to meet in order to be included in the sample: business ownership, size and CEO tenure.

For the first condition, we considered only limited companies because it is easier to obtain their financial data due to the fact that they have to publicize their financial accounts every year. Concerning the second condition, we only considered large companies, which we defined according to the number of employees (> 250), their volume of assets (> 9,495 million Euros) and their volume of business (> 18,991 million Euros), following Spanish and European regulations [7, 8 and 9]. However, we decided to include only those companies with a minimum of 400 employees, since the median of the number of employees of large Spanish companies during the analyzed period goes from 454 (in 2001, the first year of the period under consideration) to 639 (in 2014). Therefore, we have taken the number of 400 employees as a stable figure throughout the entire period studied.

Finally, for the financial data and tenure of their CEOs, we accessed the SABI database (Analysis System of Iberian Balance Sheets), which is a source of economic and financial information on Spanish and Portuguese companies and also provides the name of their CEOs during the period under consideration. The source of information of the SABI database comes from those legal documents required by the Spanish Mercantile Registry.

For this study, we compiled a list of 573 companies satisfying all required conditions, but we excluded financial companies because their balance sheets have a structure different from non-financial companies. The response rate of our survey was 16%, which is in line with the rate used as a benchmark in the literature (rate of 11% from CEOs and CFO trait surveys [5]; rates of 10% and 16% from CEO opinion surveys [10, 11], respectively). Nevertheless, due to the significant non-response rate of our survey, we have decided to use a stratified random sampling with optimal allocation in order to reduce sampling errors.

The stratified random sampling with optimal allocation is defined as a function of the number of sectors and their corresponding sizes selected from all companies which meet our criteria. The sampling size is given by the following formula:

$$n=\frac{{\left(\sum_{h=1}^{L}W_{h}S_{h}\right)}^{2}}{\frac{e^{2}}{k^{2}}+\frac{1}{N}\sum_{h=1}^{L}W_{h}S_{h}^{2}},$$

where *L* is the number of strata, *N* is the size of the selected population, *W*_*h*_ = *N*_*h*_/*N* is the weight of the population in the *h*-th stratum (*N*_*h*_) on the total population, *S*_*h*_ the quasi-standard deviation of the *h*-th stratum, *e* is the error, and *k* is the value corresponding to a given level of confidence. The implementation of the former formula gives a sampling size of 114 individual companies.

To calculate the size of each stratum (denoted by *n*_*h*_), we have applied the following formula:

$$n_{h}=\frac{nN_{h}S_{h}}{\sum_{h=1}^{L}N_{h}S_{h}}.$$

The size of the target population has been fixed at 573 companies. In order to organize the intermediate calculations to obtain the size of each sampling stratum, it is necessary to divide the 573 companies by segments (represented by *N*_*h*_) and the quasi-standard deviation (denoted by *S*_*h*_) shown by the six chosen sectors into which we have divided the economic activity (S1 Table).

The results derived from second equation, by using the intermediate results included in S1 Table, have been shown in S2 Table. This calculation has been performed at a 95% confidence level. These values have been labeled as “Theoretical values of *n*_*h*_”. However, because of its limitations when administering the questionnaire, the final number of companies consulted in each stratum appears under the label “Real values of *n*_*h*_”. The final column of S2 Table shows the deviations of real with respect to theoretical values of each sampling stratum.

### Time horizon

The time horizon of this study is the period between 2001 and 2014, hereinafter the “period 2001–2014” which in fact covers two clearly differentiated sub-periods. The first is a period of expansion and covers the years 2001 to 2007. In contrast, the second is a period of recession, from 2008 to 2014, because in the second quarter of 2008 Spain went into a recession which finalized in 2014. Macroeconomic data show some evidence of significant changes in the Spanish economy in 2008. For example, the Gross Domestic Product (GDP) diminished during 2008 and 2009 (−3.6%) with six consecutive quarters of decline [12] but increased by 1.4% in 2014 [13]; employment declined rapidly from 2008 to 2012 [14]; domestic demand fell sharply (7.6%) from 2008 to 2010, whereas in the Eurozone it only declined by 1.6%. Consequently, productivity also decreased and households reduced their rates of savings to their lowest level due to investment in fixed capital (housing) and its consequent debt (130% of their gross disposable income (GDI)) [15].

### Variables and measurement

In this study, some qualitative and quantitative data have been used to test the hypothesis. Qualitative data analyze the personal traits of CEOs of the Spanish companies under consideration. The source of qualitative data is an online questionnaire which we conducted. The questionnaire contains four main parts and was built in Google Form. In the first part we introduced the aim of the questionnaire and also requested some personal CEO information, such as sex, age, education and position in the company, and company information, such as company size and sector. The second and third parts were the psychometric tests (LOT-R and DOSPERT) and the fourth part referred to the law of data protection.

To conduct the questionnaire, we first contacted the companies included in the sample and sent them the questionnaire by e-mail in the winter of 2013. Six months later, we re-sent the questionnaire to those companies which had not responded to the first submission. Furthermore, to ensure that the CEO personally completed the questionnaire, we employed two specific mechanisms. A personal question was asked in the first part, such as the university where the CEO studied, and this information was verified through the CEO’s profile in LinkedIn (if available). The second mechanism was to send the survey directly to the CEO’s e-mail address in order to avoid a reply from a secretary or assistant.

In contrast, the quantitative information was about the financial ratios of the companies in which the CEOs were employed. The source of these financial ratios was the SABI database.

#### Qualitative variables

The personal traits considered in the study refer to the personality of CEOs and heuristic considerations, which are usually applied when taking decisions involving an element of risk; that is, dispositional optimism, attitude towards risk and affect heuristic.

The dispositional optimism is a polar unidimensional construct which can be defined as a general and positive/negative expectation about the future [16, 17]. Furthermore, this trait is based on the model of behavioral self-regulation which assumes that, when complications arise, positive expectations increase the effort of achievement, whilst negative expectations lead to inaction [18]. Consequently, when a situation is difficult or stressful, dispositional optimism acts as a problem-focused strategy [19, 20].

To measure CEO’s optimism, a psychometric test was included in the survey, more specifically, the Life Orientation Test-Revisited (LOT-R) because it has been used in different fields, such as psychology [21], medicine [22], and corporate finance [5].

The LOT test was designed by Scheier and Carver [16] and then revised (LOT-R) by Carver et al. [17] resulting in a brief and easy test which has a clear, direct and unequivocal interpretation. It consists of ten items three of which have positive orientation, for example, “In uncertain times, I usually expect the best”; a further three have a negative orientation, for example, “I hardly ever expect things to go my way”; and the remainders are filling items such as “It is easy for me to relax”. The questionnaire uses a Likert scale [23] ranging from 0 to 4 to weight the responses: I agree a lot, I agree a little, I neither agree nor disagree, I disagree a little, and I disagree a lot. In this study, the Spanish version of LOT-R [24] was employed.

In order to assess the level of optimism of each CEO, it is necessary to sum up the scores corresponding to positive and negative items (the score of a negative item is taken in absolute value). Following the cut-off value proposed by Graham et al. [5], in a financial framework, a CEO is classified as highly optimistic if the result of the test scores 18 or above, and low optimistic if it is below 17.

On the other hand, the attitude towards risk is defined, from a psychological point of view, as a chosen state of mind with respect to those uncertainties which could have a positive or negative effect on objectives [25]. In spite of the fact that this attitude is considered to be a personality trait [26], it is not always consistent with different domains and situations [27]. On the other hand, from a financial point of view, risk attitude can be embedded in a risk-return framework in the context of risky decisions. This means that the preference for risky options is assumed to reflect a trade-off between the expected benefit of an option, equal to the expected value, and its level of risk [28]. By considering both views, a psychological risk-return model considers the perceived riskiness as a variable which is different for each person, content and context [28]. Furthermore, the basic attitudes towards risk are defined as risk-averse, risk-tolerant and risk-seeking.

In order to quantify and assess a person’s attitude towards risk, different methods have been designed. Charness et al. [29] classify them from complex to simple, and state that, whilst complex methods are used to estimate the parameters of a model, simple methods are usually easier to understand by participants. Some examples of simple methods are lotteries with single or multiple decisions, which can be considered as an investment decision, and a survey in which participants are asked to express their own risk preferences through hypothetical-risk questions. Moreover, methods for eliciting risk attitude can be incentivized or not [30].

The method to elicit and assess the attitude towards risk depends on the question which the research aims to answer, as well as the characteristics of the sample population [30]. In this case, due to the characteristics of the population (busy CEOs) and a lack of resources and time, an incentivized survey method was chosen. In addition, an online questionnaire is also easier to understand by CEOs.

A Spanish version of the Domain-Specific Risk-Attitude Scale (DOSPERT) [31] was chosen to conduct the survey. This is a risk-taking scale developed by Blais and Weber [28] in a risk-return framework which allows us to elicit and assess both risk-taking and perceived-risk attitude in five domains (ethical, financial (gambling and investment), health/safety, social, and recreational). It includes three separate response scales: risk-taking, risk-perceptions, and expected benefits; and each scale has six different items for every domain. In order to evaluate each item, the likelihood of a risk being taken is measured from “extremely unlikely” to “extremely likely” by using a Likert scale. To measure the perceived risk level of domain items, the Likert scale ranges from “not at all risky” to “extremely risky”. Finally, it measures the benefits expected from the items ranging from “no benefits” to “great benefits”.

In this study only the financial domain was assessed. The items in this domain were about gambling, such as “Betting a day’s income at the horse races”, and about investments such as “Investing 5% of your annual income in a very speculative stock”. Gambling items were considered because a CEO’s attitude to gambling is related to some corporate decisions such as acquisitions [32] and mergers [5].

In order to calculate the result of each scale, it is necessary to sum up the rating scores of all items in a domain (see the website https://sites.google.com/a/decisionsciences.columbia.edu/dospert/scoring-instructions). With these sums and in order to calculate the coefficient of each independent response scale, the attitude towards risk can be obtained by regressing the results of risk-taking scales on the results of expected benefits and of risk-perception scales. The sign of the risk-perception coefficient indicates the attitude towards risk, where a positive coefficient indicates a risk-seeking behavior and a negative coefficient means risk-aversion behavior:

Risk‐taking(X)=a(ExpectedBenefit(X))+b(PerceivedRisk(X))+c

The affect heuristic is a mental short-cut which labels objects and events in people’s minds according to its affect. Fischhoff et al. [33] suggest that, in the decision-making process, individuals consult consciously or unconsciously an affect pool which contains all affect tags associated with the representation. Furthermore, this heuristic is unconsciously applied to decisions and to risk perception.

In the field of finance, the relation between risk and benefits is positive; in contrast, in people’s minds this relation is negative [34]. Alhakami and Slovic [34] found that the inverse relation between perceived risk and perceived benefits of financial activities is linked to the positive or the negative affect associated with them as measured by polar scales (good/bad, pleasant/awful, and so forth). Consequently, if the feelings towards an activity are favorable, people judge the risk as low and the benefits as high, and vice versa.

The data of both the risk-perception and the expected benefits from the DOSPERT test have been used to measure the CEOs’ affect heuristic. If the expected-benefit rate is low (high) and the risk-perception rate is high (low), they use negative (positive) affects to evaluate the risk in financial decisions. In other cases, they do not give way to their feelings.

In this paper, the three personal traits are considered as dichotomous variables. Table 1 shows the value for each variable.

**Table 1:** Qualitative and quantitative variables, values and definition.

**Qualitative variables**	**Value**
Dispositional optimism	0 –Low optimism 1 –High optimism
Attitude towards risk	0 –Risk propensity 1 –Risk aversion
Affect Heuristic	0 –Negative affect 1 –Positive affect
**Quantitative variables**	**Definition**
Financial leverage	(Current liabilities + Long-term liabilities) / (Total liabilities + Equity)
Long-term debt	Long-term liabilities / (Total liabilities + Equity)
Short-term debt	Current liabilities / (Total Liabilities + Equity)
Equity	Equity / (Total liabilities + Equity)

#### Quantitative variables

The quantitative variables are a set of financial ratios which represent the financial structure of a company (Table 1). Whilst financial leverage is the sum of all the debts of a company, equity is the amount of capital contributed by the owners. Debt is also divided according to its repayment term, into long-term or short-term. On the other hand, we have considered as the financial structure of companies the leverage of companies and its components, long-term and short-term debt, because bank debt is the main source of financing for large Spanish companies.

### Organization data and statistical tests

Due to the large amount of qualitative and quantitative data, it was decided to use panel data to apply statistical tests. The advantage of panel over cross-section regression is that the second methodology allows a careful modeling of unobservable data, which can be divided into two components: between-company and within-company. The heterogeneity cannot be detected either with the analysis of time series or with cross-sectional techniques. Additionally, panel data also provides more variability and informative data and presents less collinearity among the variables. Furthermore, it permits a greater degree of freedom and is more efficient than cross-section or time-series data. In contrast, it has some limitations, such as problems with the design and data collection and distortions of measurement errors [35, 36].

On the one hand, we have applied a univariate analysis of qualitative and quantitative variables to describe the data. For qualitative or independent variables, we have analyzed the levels or scales of each trait. For quantitative dependent variables, we have analyzed the data for the period 2001–2014 by distinguishing between economic periods.

As for the applied test, we have implemented a correlation test to observe the association of CEOs’ personal traits with the financial structure of companies. To observe the associations from a broader perspective, we considered the entire period 2001–2014.

On the other hand, to analyze the relation between CEOs’ traits and the financial structure, we have applied three random-effects regression analyses, one for each dependent variable. We have chosen this type of regression for two reasons. On the one hand, the Hausman test (X^2^ = 19.41; *p* > 0.020) rejects the null hypothesis that the error term is not correlated with regressors, and so suggesting the use of a fixed-effects regression model; however, this model omits CEOs’ personality traits because they are time-invariant. Consequently, we have been forced to run random-effects panel regressions. On the other hand, our sample has been taken from a large number of companies: the variation between them is assumed to be random and uncorrelated with the independent variables. That is to say:

$$cov\left(X_{itk}^{\prime },\mu_{i}\right)=0;\;t=1,\ldots ,T;\;k=1,\ldots ,K.$$

We have applied the Generalized Least Square (GLS) method and considered the Panel-Corrected Standard Error (PCSE) estimation with autocorrelation-correction so as to robust disturbances which are assumed to be heteroscedastic, contemporaneously cross-sectionally correlated and autocorrelated of type AR(1).

To examine the effect of the country’s economic situation on the relation between CEOs’ personal traits and the financial structure of large Spanish companies, firstly we applied Chow’s test to examine the presence of a structural break between 2007 and 2008. After that, we have added to the regression analysis a continuous variable, called “economic period”, which represents the two time intervals included in the period 2001–2014. We have used the Spanish GDP of each period as a proxy and also added three interaction variables. The interactions are the product of each CEOs’ personal trait and the variable “economic period”. They allow us to examine whether the economic situation affects (moderates) the aforementioned relations. Subsequently, we have carried out a further analysis of these interactions. To do this, we have plotted the interaction slopes on graphs in order to observe the direction and strength of the effect of the economic situation on each trait. We have also applied the Johnson-Neyman technique [37] by obtaining the confident intervals of the relation for each economic period. This technique allows us to observe in which economic period the moderating effect on the relationship is statistically significant.

### Limitations

It is assumed that the dispositional optimism, the attitude towards risk and the affect heuristic are stable traits over time. Although we measured CEOs’ personal traits in the period of economic contraction, it is possible to extrapolate the result to the period of economic expansion.

Despite the fact that our obtained response rate is in line with the usual rate considered in the literature, the results of this study are assumed to be applied only to the selected sample.

## Results and discussion

This section tries to present and discuss the main results. Firstly, CEOs’ personal traits and the financial structure of large Spanish companies have been defined. Secondly, the relations between personal traits of executives and debt during the period 2001–2014, and under the influence of the contraction or expansion economic period, have been identified and compared.

### Personal characteristics of CEOs

This subsection defines CEOs’ personal traits by analyzing the demographic and psychological data derived from the answers obtained from the aforementioned online questionnaire.

The results show that, within the sample, 50-year-old men with a Higher Education qualification occupy the position of CEO in large Spanish companies. The minimum CEO age is 35 years and the maximum 65 years. As for qualifications, 40% of them had reached post-graduate level. On the other hand, women only represent 9.6% of respondents (S3 Table).

Regarding the psychological characteristics (S4 Table), CEOs of large Spanish companies belonging to the sample exhibit a high optimism (68.69%), as shown by the psychometric tests (LOT-R score 20.51, sd. 1.74). In contrast, this result gives a lower “highly optimistic” percentage figure than that obtained for executives of large US companies (80.2%; LOT-R score 20.34, sd. 3.50) [5].

As for CEOs’ attitude towards risk, the results from the psychometric test indicate that 60.86% of CEOs of large Spanish companies in the sample show an adverse attitude towards risk in financial activities (gambling and investment) (S4 Table). More specifically, they show more disposition to engage in investment than in gambling tasks. They perceived a level of risk higher in gambling than in investment tasks, but their expected benefits are lower (see S4 Table). In large US companies, only 9.8% of CEOs show a low level of risk tolerance [5].

As for affect heuristic, the affect which CEOs associate with financial tasks may determine their evaluation of financial activities. Whilst executives’ valuation of gambling activities is likely to be determined by a negative affect (81.74%), the assessment of investment activities may be conditioned by a positive affect (69.57%) (S4 Table).

### Characteristics of the financial structure of companies

This subsection describes the financial structure of large Spanish companies for the period 2001–2014 and for the expansion and contraction economic periods.

The results derived from the univariate analysis report that, over the period 2001–2014, the composition of the financial structure of Spanish companies was mainly debt (59%). Specifically, large Spanish companies preferred financing their operations with short-term debt (77%) rather than with long-term debt.

Whilst equity remained equal during the time horizon, there were significant differences in the composition of the financial leverage in each economic period (Table 2). Specifically, companies decreased the short-term banking debt (−5.51%) and increased the long-term banking debt (3.85%).

**Table 2:** Capital structure.

	2001–2007		2008–2014	
	Mean	Std. Dev.	Mean	Std. Dev.
Financial leverage	0.6003	0.2193	0.5930	0.2454
Long-term debt	0.1293	0.1477	0.1678	0.1673
Short-term debt	0.4709	0.2234	0.4158	0.2192
Equity	0.4006	0.2014	0.4071	0.2217

### The relation between CEOs’ personal traits and the financial structure of large Spanish companies

This subsection shows what CEOs’ personal traits are related with the financial structure of large Spanish companies in the long term. Additionally, the relations are compared to the findings given in the existing literature.

#### The relation between CEOs' personal traits and the financial leverage of companies in the long term

The results from statistical tests suggest that, among the executives’ traits, only the positive affect has a relation with the financial leverage of large Spanish companies in the long term (Table 3). The test of correlation shows a positive association between both variables, and the random-effects regressions confirm such relation (0.0719, *p* < 0.05). When CEOs handle financial leverage affairs, it is probable that they expect higher profits and a low perceived risk when financing with debt.

**Table 3:** Correlation and random-effects regression models.

	Financial Leverage		Long-Term Debt		Short-Term Debt	
	Correlation	Random-Effects Regression	Correlation	Random-Effects Regression	Correlation	Random-Effects Regression
High Optimism	−0.0413	−0.0141 (0.0378)	0.1651*	0.0614* (0.0244)	−0.0569**	−0.0241 (0.0378)
Risk-Averse	−0.0929**	−0.0466 (0.0371)	−0.1219**	−0.0455* (0.0239)	−0.0339	−0.0162 (0.0371)
Positive Affect	0.1248**	0.0719* (0.0433)	−0.0809**	−0.0272 (0.0279)	0.0874**	0.0474* (0.0433)
Constant	-	0.5793 (0.0495)	-	0.1717 (0.0319)	-	−0.4387 (0.0496)
Number of observations	-	1582	-	1582	-	1582

Note: The Generalized Least Square (GLS) method and the Panel-Corrected Standard Error (PCSE) estimation with autocorrelation-correction to robust disturbances which are assumed to be heteroscedastic, contemporaneously cross-sectionally correlated and autocorrelated of type AR(1) was applied.

** significance code .01

* significance code .05.

On the other hand, CEOs’ risk aversion and high optimism are not related with the leverage of companies. Despite the fact that CEOs’ risk aversion attitude is negatively associated with the leverage, the result of the regression suggests that this trait is not statistically significant (Table 3 column Financial Leverage).

#### The relation of CEOs' personal traits and the long and short-term debt of companies in the long term

The results reveal that CEOs’ high optimism and risk aversion traits have a relation with the long-term debt of large Spanish companies (Table 3). For high optimism, we expected that this trait would be associated with short-term [5] [6]; in contrast, we found that it is positively related with long-term debt (0.0614, *p* < 0.05). Thus, it is probable that executives create positive expectations for their company when financing with long-term debt. As for executives’ risk aversion, Graham et al. [5] suggest that this trait is not related with debt; however, we found a negative relationship between this attitude and the long-term debt (−0.0455, *p* < 0.05). Hence, it is possible that CEO perceive that funding the company with long-term debt entails a high level of risk, despite the fact that this term of debt is less risky. Considering that both personal traits are correlated (S5 Table), CEOs may create positive expectations for their company when funding with a perceived safety level of long-term debt. Possibly, they try to balance both personal traits when dealing with a long-term-debt funding option.

On the other hand, the affect heuristic of executives is the only personal trait related with the short-term debt of large Spanish companies (Table 3). As expected, there is a positive relationship with the short-term debt (0.0474, *p* > 0.05). Therefore, it is likely that they expect high benefits and perceive a low risk of funding with short-term debt.

### The relation between CEOs’ personal traits and the financial structure of large Spanish companies under the influence of economic periods

This subsection reveals the relation between CEOs’ personal traits and the financial structure of large Spanish companies under the moderating effect of the economic situation in the country.

#### The relation of CEOs' personal traits and the financial leverage of companies under the influence of the economic situation

**Fig 1:**
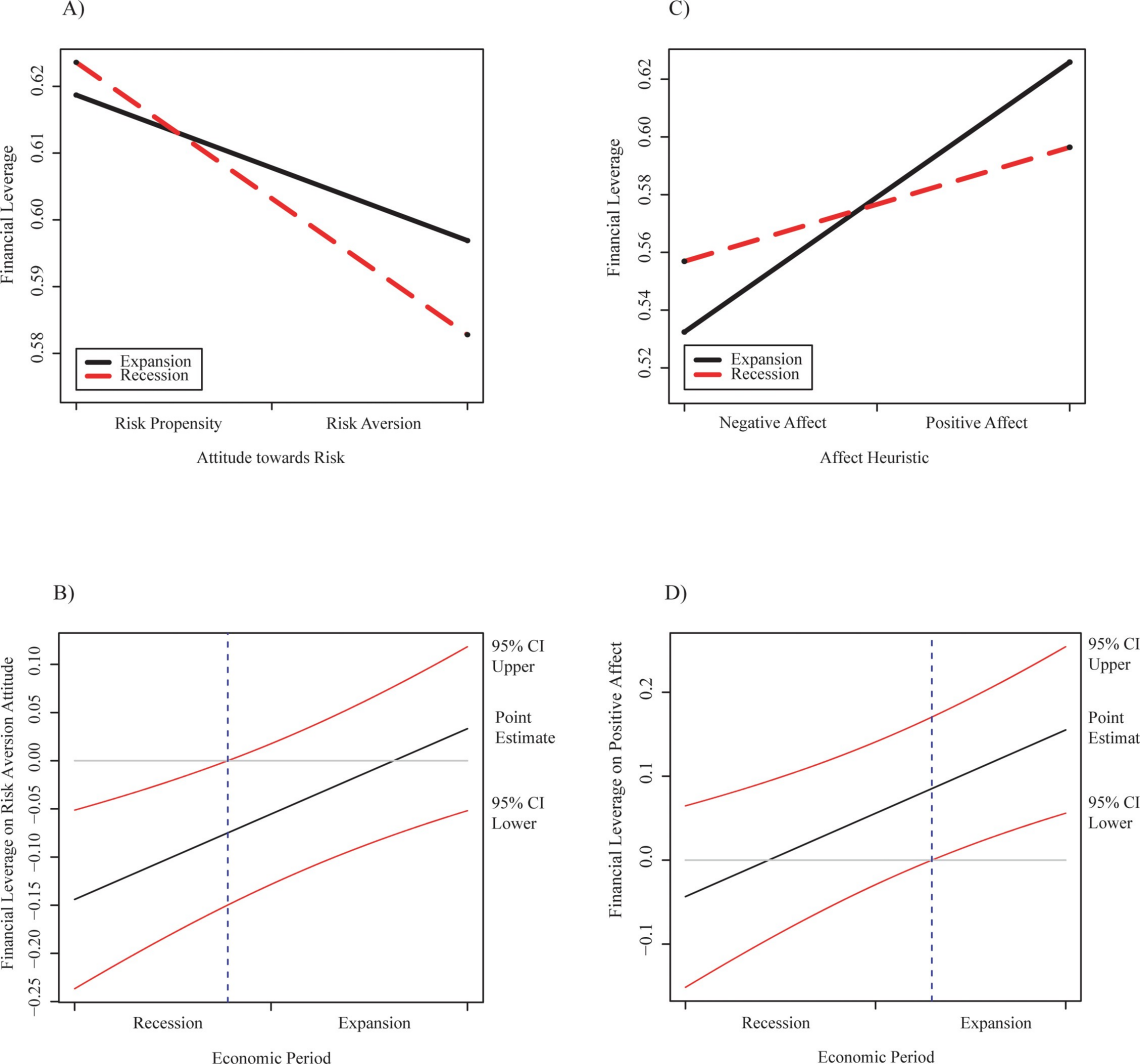
Interactions and confidence intervals graphs of the relation of traits and financial leverage. A) Graph of simple slopes of risk attitude of CEOs on total debt in function of the economic situation. B) Confidence intervals of risk attitude of CEOs on total debt for the two economic periods. C) Graph of simple slopes of affect heuristic on total debt in function of the economic situation. D) Confidence intervals of positive affect heuristic of CEOs on total debt for the two economic periods.

Our results reveal that the relation of CEOs’ risk aversion with the leverage of companies is significantly influenced by the added pressure of a period of recession at national level. This relation does not appear in our early regression analysis but, when the executives’ risk aversion interacts with the economic situation, the relation becomes significant (0.0084, *p* < 0.05) (see Table 4). In addition, a simple analysis indicates that both economic periods exert a strong moderating effect on this relationship (Fig 1A), but the effect on the relation is only statistically significant in the period of recession (Fig 1B).

**Table 4:** Random-effects regression models in expansion and contraction economic periods.

Random-effects regressions during a period of economic expansion and contraction			
	Total Debt	Long-Term Debt	Short-Term Debt
High Optimism	−0.0120 (0.0378)	0.0671* (0.0244)	−0.0254 (0.0375)
Risk Adverse	−0.0577 (0.0371)	−0.0488* (0.0239)	−0.0268 (0.0369)
Positive Affect	0.0598* (0.0433)	−0.0362 (0.0279)	0.0504 (0.0430)
Economic Situation	−0.0081* (0.0034)	−0.0065* (0.0030)	0.0065 (0.0095)
High Optimism*Economic Situation	−0.0016 (0.0026)	−0.0043 (0.0023)	−0.0011 (0.0004)
Risk Adverse*Economic Situation	0.0084* (0.0025)	0.0025 (0.0022)	0.0065* (0.0002)
Positive Affect*Economic Situation	0.0092* (0.0030)	0.0069* (0.0026)	−0.0017 (0.0001)
Constant	0.5899 (0.0495)	0.1803 (0.0319)	0.4322 (0.0492)
Number of observations	1,582	1,582	1,582

Note: The Generalized Least Square (GLS) method and the Panel-Corrected Standard Error (PCSE) estimation with autocorrelation-correction to robust disturbances which are assumed to be heteroscedastic, contemporaneously cross-sectionally correlated and autocorrelated of type AR(1) was applied.

** Significance code .01

* Significance code .05.

Our findings also suggest that the relationship of CEOs’ positive affect with the leverage of companies is significant when it is strongly moderated by the expansion period. When executives’ risk aversion interacts with the economic situation, the relation with the financial leverage becomes statistically significant (0.0092, *p* < 0.05). Furthermore, the simple slope analysis suggests that the moderating effect of both economic periods on the relationship is strong (Fig 1C) and the confidence intervals indicate that the effect on the relation is only statistically significant in the economic period of expansion (Fig 1D).

It is likely that, during the expansion period, CEOs expect more benefits and perceive less risk of funding their companies with debt than with equity. Nevertheless, under the contraction period, executives may reduce the financial leverage of their companies to avoid taking more risks.

#### The relation between CEOs' personal traits and the long- and short-term debt of companies under the influence of economic periods

**Fig 2:**
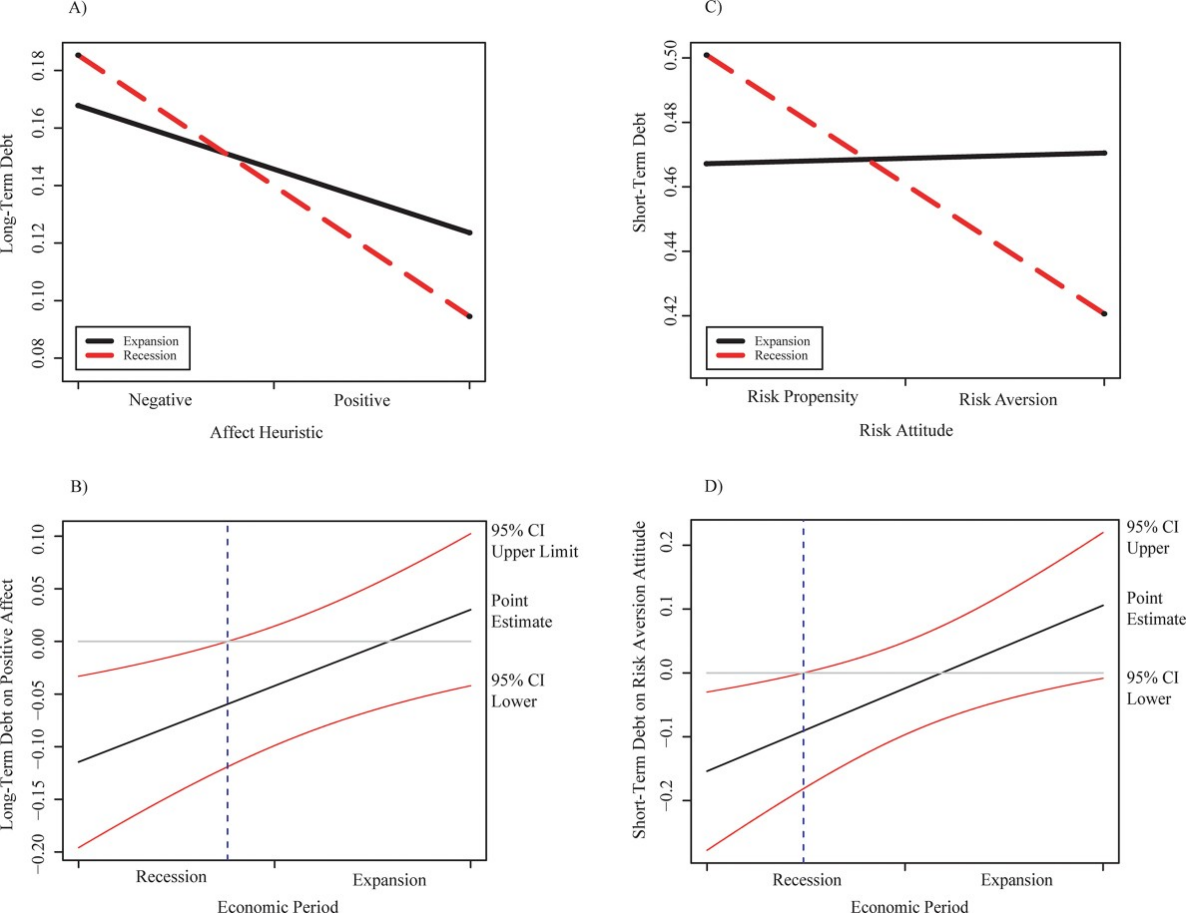
Interactions and confidence intervals graphs of the relation of traits and long-term and short-term debt. A) Graph of simple slopes of positive affect heuristic of CEOs on long-term debt in function of the economic situation. B) Confidence intervals of positive affect of CEOs on long-term debt for the two economic periods. C) Graph of simple slopes of adverse risk attitude of CEOs on short-term debt in function of the economic situation. D) Confidence intervals of adverse risk attitude of CEOs on short-term debt for the two economic periods.

Our findings indicate that the relationship of CEOs’ positive affect with long-term debt is significant when the recession economic period strongly moderates it. This relationship does not appear in our early regression analysis. In contrast, when executives’ positive affect interacts with the economic period, the relation becomes statistically significant (0.0069, *p* < 0.05), as shown in Table 4. A simple analysis suggests a strong moderating effect of both economic periods on this relationship (Fig 2A). Additionally, the confidence intervals indicate that the effect on the relation is statistically significant in the recession economic period (Fig 2B). On the other hand, our findings also reveal that the economic situation does not moderate the relations of CEOs’ high optimism and the risk aversion attitude to long-term debt.

Our results (see Table 4) suggest that the recession economic period strongly moderates the positive relationship of CEOs’ risk aversion attitude with the short-term debt. This relationship does not appear in our early regression analysis; however, when CEOs’ risk aversion interacts with the economic period, the relation becomes statistically significant (0.0065, *p* < 0.05). Fig 2C shows that the economic period produces a strong moderator effect on the relationship but the effect on the relation is statistically significant only in the recession period (Fig 2D).

Despite CEOs’ positive expectation of funding with long-term debt, the level of debt which executives perceive as acceptable to avoid taking high risks is lower in the contraction than in the expansion economic period. Furthermore, during the contraction period, they may perceive short-term debt as a more risky debt option than in an expansion period, and so prefer to reduce it considerably.

## Conclusion

We have shown that there are several links between CEOs’ personal traits and the financial leverage of large private Spanish companies for the analyzed period 2001–2014. In contrast, the meaning of these relationships is incomplete if the economic situation of the period is not taken into account. The relation of executives’ traits with the financial leverage and the short-term debt of their companies is moderated by the economic situation, specifically the contraction period. Nevertheless, not all relationships between CEOs’ traits and the long-term debt are moderated. Indeed, the awareness of these relations will help to understand the financial decisions of large Spanish companies.

## Supporting information

S1 Table Distribution and quasi-standard deviation of economic sectors. (DOCX)

S2 Table Real values and theoretical values of each stratum. (DOCX)

S3 Table CEO profile. (DOCX)

S4 Table Results from the psychometric tests. (DOCX)

S5 Table Correlations between CEOs’ traits. (DOCX)
