# Supplementary material for: Economic situation, the key to understanding the links between CEOs’ personal traits and the financial structure of large private companies
Source: PLoS One. 2019 Jul 18;14(7):e0218853. doi: 10.1371/journal.pone.0218853 (PMC6638866; doi:10.1371/journal.pone.0218853)
Supplement: S1 Table — (DOCX) [file pone.0218853.s001.docx]

**S1 Table. Distribution and quasi-standard deviation of economic sectors**

| Name of stratum | *N_h_* | *S_h_* |
| --- | --- | --- |
| Oil and energy | 24 | 17.76 |
| Basic materials, industry and building | 125 | 20.65 |
| Consumer goods | 99 | 28.60 |
| Consumer services | 283 | 23.27 |
| Financial and real estate services | 6 | 18.18 |
| Technology and telecommunications | 36 | 25.02 |
| *N* = | 573 | 28.84 |
