# Supplementary material for: Economic situation, the key to understanding the links between CEOs’ personal traits and the financial structure of large private companies
Source: PLoS One. 2019 Jul 18;14(7):e0218853. doi: 10.1371/journal.pone.0218853 (PMC6638866; doi:10.1371/journal.pone.0218853)
Supplement: S2 Table — (DOCX) [file pone.0218853.s002.docx]

**S2 Table. Real values and theoretical values of each stratum**

| **Name of stratum** | **Real values of *n_h_*** | **Theoretical values of *n_h_*** | **Deviation** |
| --- | --- | --- | --- |
| Oil and energy | 6 | 4 | +2 |
| Basic materials, industry and building | 23 | 22 | +1 |
| Consumer goods | 25 | 24 | +1 |
| Consumer services | 51 | 56 | −5 |
| Financial and real estate services | 1 | 1 | 0 |
| Technology and telecommunications | 8 | 8 | 0 |
| *N =* | 114 | 114 | 0 |
