# Supplementary material for: Economic situation, the key to understanding the links between CEOs’ personal traits and the financial structure of large private companies
Source: PLoS One. 2019 Jul 18;14(7):e0218853. doi: 10.1371/journal.pone.0218853 (PMC6638866; doi:10.1371/journal.pone.0218853)
Supplement: S3 Table — (DOCX) [file pone.0218853.s003.docx]

**S3 Table. CEO profile**

|  | **Number** | **Percentage** |
| --- | --- | --- |
| Male | 113 | 90.40 |
| Master’s degrees | 113 | 40 |
| High Optimism | 113 | 68.69 |
| Risk Aversion | 113 | 60.86 |
| Positive Affect | 113 | 81.73 |
