# Supplementary material for: Economic situation, the key to understanding the links between CEOs’ personal traits and the financial structure of large private companies
Source: PLoS One. 2019 Jul 18;14(7):e0218853. doi: 10.1371/journal.pone.0218853 (PMC6638866; doi:10.1371/journal.pone.0218853)
Supplement: S4 Table — (DOCX) [file pone.0218853.s004.docx]

**S4 Table. Results from the psychometric tests**

| **Variables** | **Level** | | **Task** | | **Mean** | **Stand. Dev.** | **Min.** | **Med.** | **Max.** |
| --- | --- | --- | --- | --- | --- | --- | --- | --- | --- |
| Optimism | High | | | | 20.51 | 1.74 | 18 | 21 | 24 |
|  | Medium | | | | 15.63 | 1.35 | 13 | 16 | 17 |
| Risk attitude | Risk taking | Gambling | | | 4.11 | 2.98 | 3 | 3 | 20 |
|  |  | Investment | | | 13.44 | 3.73 | 3 | 14 | 20 |
|  | Risk perception | Gambling | | | 16.21 | 5.89 | 3 | 18 | 21 |
|  |  | Investment | | | 10.36 | 3.05 | 3 | 10 | 18 |
|  | Expected benefits | Gambling | | | 6.23 | 4.67 | 3 | 4 | 21 |
|  |  | Investment | | | 13.03 | 2.89 | 6 | 13 | 19 |
| Affect heuristic |  | Gambling | | Positive affect (%) | 18.26 | - | - | - | - |
|  |  |  |  | Negative affect (%) | 81.74 | - | - | - | - |
|  |  | Investment | | Positive affect (%) | 69.57 | - | - | - | - |
|  |  |  |  | Negative affect (%) | 30.43 | - | - | - | - |
