# Supplementary material for: Economic situation, the key to understanding the links between CEOs’ personal traits and the financial structure of large private companies
Source: PLoS One. 2019 Jul 18;14(7):e0218853. doi: 10.1371/journal.pone.0218853 (PMC6638866; doi:10.1371/journal.pone.0218853)
Supplement: S5 Table — (DOCX) [file pone.0218853.s005.docx]

**S5 Table. Correlations between CEOs’ traits**

|  | High Optimism | Risk Aversion | Positive Affect |
| --- | --- | --- | --- |
| High Optimism | 1 |  |  |
| Risk Aversion | 0.0828** | 1 |  |
| Positive Affect | -0.0282 | 0.0653** | 1 |

Note: ** significance codes .01; * significance codes .05
